# The combination of fatigue with the serum GCSF improves the performance of serological screening for frailty: Fatigue with GCSF improves the performance of serological screening for frailty

**DOI:** 10.3724/abbs.2025007

**Published:** 2025-02-25

**Authors:** Jiaming Yu, Jie Chen, Xueying Ji, Yixuan Qiu, Yan Zhang, Jiaofeng Wang, Xiangqi Li, Chaobao Zhang, Zhijun Bao

**Affiliations:** 1 Shanghai Key Laboratory of Clinical Geriatric Medicine; Department of Geriatric Medicine Huadong Hospital Fudan University Shanghai 200040 China; 2 National Clinical Research Center for Aging and Medicine Huashan Hospital Fudan University Shanghai 200040 China; 3 Department of Endocrinology and Metabolism Gongli Hospital Shanghai 200135 China

Frailty is a common geriatric disease characterized by accelerated aging and the loss of biological reserves across multiple organs [1]. Approximately 10% of people aged 65 years and older and 25%–50% of people older than 85 years are in a frail state [2]. The increasing institutionalization, hospitalization, and mortality caused by frailty incur massive medical costs and impose a heavy health service burden [3]. For elderly individuals with chronic and/or infectious diseases, such as COVID-19, concomitant frailty can lead to extremely high mortality rates [4]. Moreover, except exercise and nutritional intervention, no effective medicine for treating frailty is available [5]. However, frailty can be prevented, and prefrailty can be reversed [1]. Therefore, effectively screening frailty in elderly individuals is a public health priority. Two main methods for assessing frailty exist: the Fried phenotype [6] and the Rockwood frailty index [7]. The Fried phenotype uses five items, namely, fatigue, weakness, slowness, low physical activity, and weight loss, whereas the Rockwood frailty index is based on the accumulation of age-related deficits. However, these two diagnostic tools are subjective, challenging to use and time-consuming, and are therefore unsuitable for simple, rapid, and extensive screening of frailty in clinical practice. Here, we propose a new strategy to address the above issue.

We first harnessed common professional databases to perform inflammatory niche analysis for plasma proteomics from normal aging and frailty patients (see the Supplementary Methods). We subsequently performed frailty screening and blood sample collection. A total of 852 elderly people were included in the study from January 2018 to August 2018. The assessments included demographic information collection, frailty evaluation ( Supplementary Figure S1), and physical and body composition tests. Blood samples for the determination of inflammatory cytokines were taken from 67 participants. The flow chart of the methods is shown in Supplementary Figure S2. We utilized ELISA to detect the expressions of inflammatory cytokines and chemokines. All blood samples were collected and centrifuged at 4°C and 2000 *g* for 20 min. The serum was aliquoted and stored properly for ELISA. Inflammatory cytokines in human sera were quantified using corresponding human ELISA kits (Shanghai Xinyu Biotechnology, Shanghai, China) according to the manufacturer’s protocols. The following markers were measured: IL1A, IL2, IL6, IL8, IL10, IL17, TNFα, IFNγ, GCSF, MCP2, CXCL1, CX3CL1, MMP7, and SOD1 ( Supplementary Table S1). All the statistical analyses were performed with Prism v.6.0 (GraphPad Software, San Diego, USA). *P* < 0.05 was considered statistically significant. The receiver operating characteristic (ROC) curve was used to evaluate the performance of all the screening tools.

Our inflammatory niche analysis revealed intriguing results when five datasets containing a large number of proteins related to normal aging and inflammation were utilized. The Venn diagrams in Figure 1 show 77 human senescence-associated secretory phenotype genes. Among these genes, 26 are positively correlated with normal aging ( Figure 1A), whereas 8 are negatively correlated with normal aging ( Figure 1B). However, neither are positively correlated with frailty ( Figure 1C), and only two genes are negatively correlated with frailty ( Figure 1D). Therefore, frailty is obviously distinct from normal aging. Given that most of these differential proteins are inflammatory factors, frailty and normal aging may involve different inflammatory niches. These results suggest that inflammatory factors may be candidates for frailty screening.

**Figure 1:**
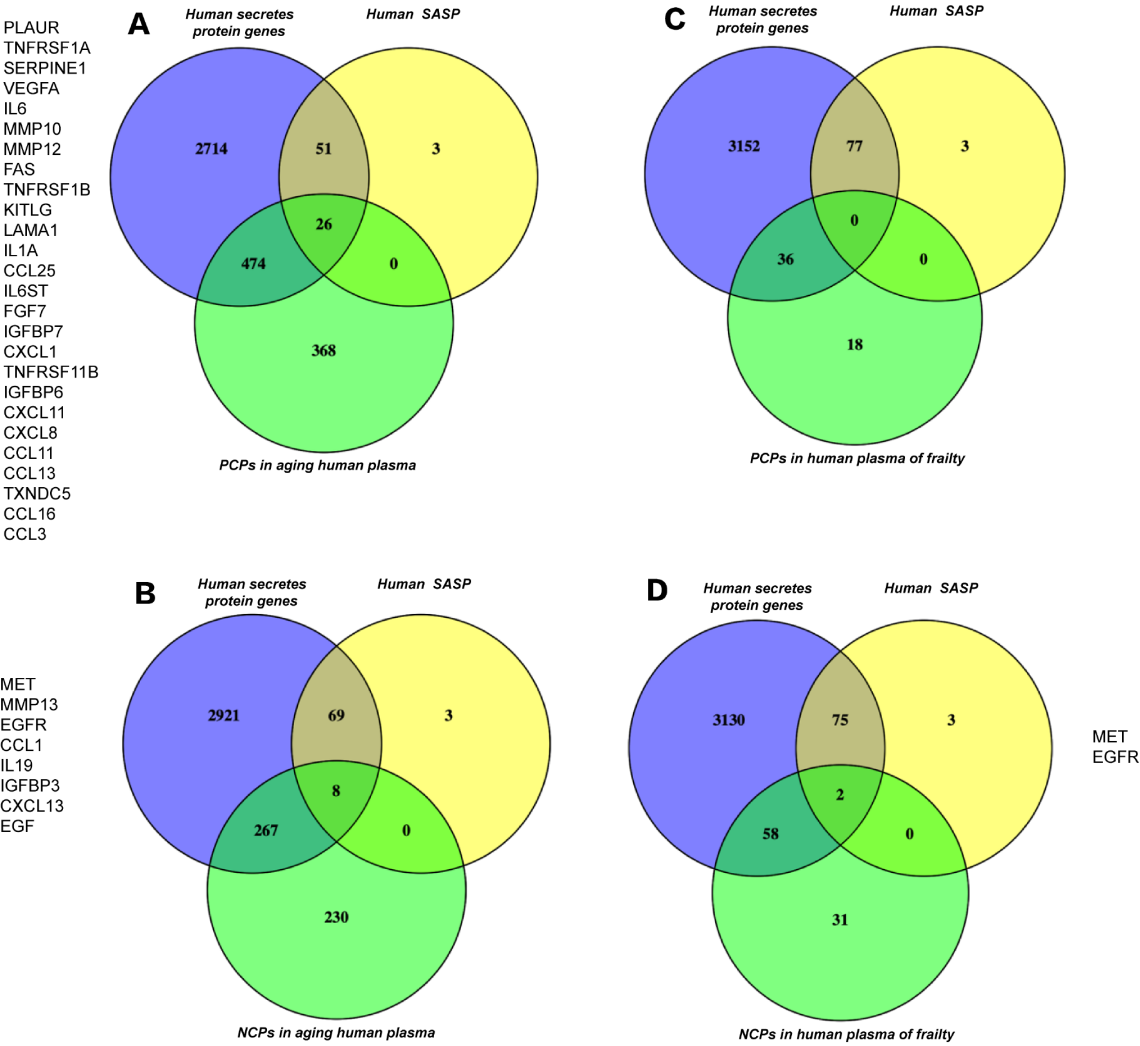
Figure 1 Frailty and normal aging involve different inflammatory niches (A) Several senescence-associated secretory phenotypes (SASPs) in human plasma are positively correlated with senescence. (B) A few SASPs in human plasma are negatively correlated with senescence. (C) No SASP in human plasma is positively associated with frailty. (D) Almost no SASP in human plasma is negatively associated with frailty. PCPs: positively-correlated proteins; NCPs: negatively-correlated proteins.

Our ELISA detection of 15 frailty-related inflammatory factors in the serum of frailty patients screened from 852 volunteers also provided valuable results. The prevalence of frailty was 7.16% (61/852), and that of prefrailty was 41.90% (357/852). The volunteers were 65.22% female and 34.78% male, with a mean age of 70.18 ± 0.73 years. As shown in Table 1, the frail and prefrail groups were significantly older than the robust group ( *P* < 0.001). We observed statistically significant differences in RASM, gait speed, CCI, SARC-F, ADL and MNA scores ( *P* < 0.05), whereas no significant differences were detected with respect to sex, WHR, grip strength or drug count among the three groups ( Table 1). A total of 67 serum samples (nonfrail, *n =*20; prefrail, *n =*28; and frail, *n =*19) were subjected to inflammatory factor screening. No significant difference in the expression of most inflammatory factors was detected between the prefrail and nonfrail groups or between the frail and nonfrail groups ( Figure 2A,B). However, the frail and prefrail groups had lower levels of IFNγ and GCSF than the nonfrail group did ( Figure 2C,D). Moreover, the TNFα level in the frail group was significantly lower than that in the nonfrail group ( Figure 2E). Interestingly, the levels of IFNγ, GCSF, and TNFα were significantly positively correlated with age in the nonfrail group and negatively correlated with age in the frail group ( Supplementary Figure S3A–C).

**Figure 2:**
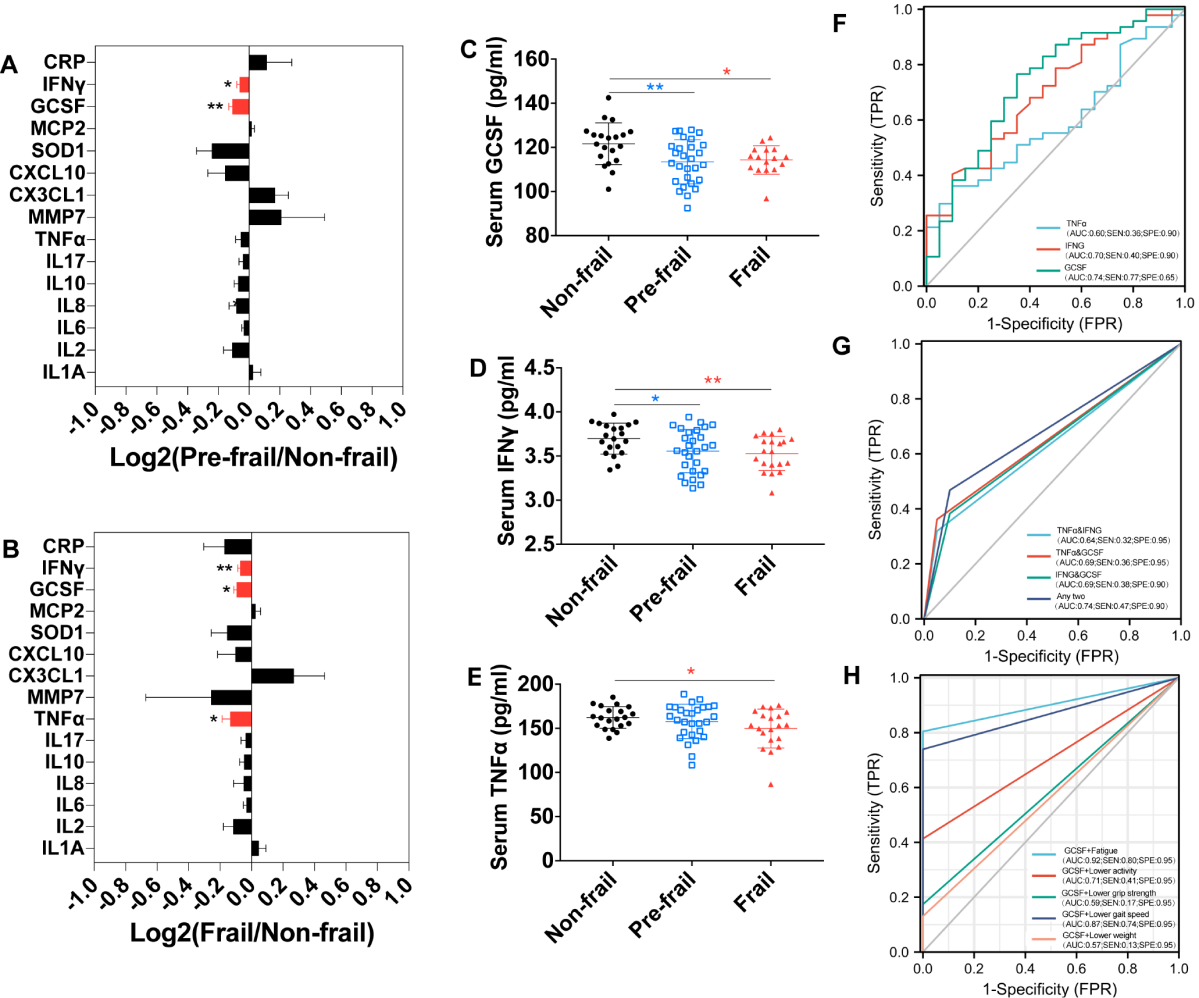
Figure 2 Assessment of inflammation-related factors as diagnostic markers for frailty (A) Screening of serum markers for prefrailty. (B) Screening of serum markers for frailty. (C) Serum GCSF expression decreased in the frail group. (D) Serum IFNγ expression was decreased in the frail group. (E) Serum TNFα expression was decreased in the frail group. (F) IFNγ, GCSF, or TNFα alone had low sensitivity for frailty and prefrailty screening. (G) The combination strategy improved the performance of serological screening candidates for frailty. (H) The combination of the GCSF score and fatigue improved the performance of frail and prefrailty screening.

**Table:** **Table 1** Baseline characteristics of the study participants by frailty status

Characteristic	Total ( *n* = 67)	Robust (NF)( *n*= 20)	Prefrail (PF)( *n*= 28)	Frailty (F)( *n*= 19)	Statistics	*P* value	Between groups
PF/NF	F/NF	F/PF
Age	70.18 ± 6.73	66.40 ± 5.18	70.21 ± 5.84	74.11 ± 7.37	7.691	0.001*	0.037*	< 0.001*	0.038*
Sex (female)	45 (65.22)	11 (55.00)	22 (78.57)	12 (63.16)	3.132	0.209	0.082	0.605	0.246
BMI (kg/m²)	23.30 ± 4.09	25.25 ± 3.71	21.90 ± 3.33	23.33 ± 4.80	4.292	0.018*	0.005*	0.131	0.221
WHR (cm/cm)	0.90 ± 0.08	0.92 ± 0.04	0.88 ± 0.08	0.91 ± 0.11	0.997	0.375	0.18	0.702	0.361
RASM (kg/m ^2^)	6.59 ± 1.64	7.59 ± 1.34	5.88 ± 1.44	6.60 ± 1.72	7.539	0.001*	< 0.001*	0.043*	0.114
Living alone	16	2 (10.00)	8 (28.571)	6 (31.579)	3.078	0.2146	0.118	0.0953	0.8249
Gait speed (m/s)	0.81 ± 0.16	0.96 ± 0.09	0.77 ± 0.14	0.70 ± 0.14	23.59	< 0.001*	< 0.001*	< 0.001*	0.064
Grip strength (kg)	24.70 (20.50–33.30)	30.3 (23.50–38.60)	23.00 (19.30–26.40)	25.30 (16.00–33.03)	5.128	0.077	0.075	0.054	0.737
CCI	0.50 (0.00–1.00)	0.00 (0.00–2.00)	1.00 (0.00–1.00)	1.00 (0.25–1.75)	5.995	0.050	0.643	0.784	0.045*
Prescription drugs	1.00 (1.00–2.00)	1.00 (0.00–2.00)	1.00 (1.00–1.00)	1.00 (1.00–2.75)	1.728	0.421	0.225	0.343	0.129
SARC-F score	0.00 (0.00–1.00)	0.00 (0.00–0.00)	0.00 (0.00–1.00)	0.50 (0.00–2.00)	13.494	0.001*	0.158	0.001*	0.121
ADL score	14.00 (14.00–14.00)	14.00 (14.00–14.00)	14.00 (14.00–14.00)	14.00 (14.00–14.00)	13.422	0.001*	1	0.006*	0.003*
MNA score	26.50 (24.50–28.00)	27.50 (26.00–28.50)	25.00 (24.50–27.00)	24.75 (21.50–27.50)	10.305	0.006*	0.027*	0.009*	0.526

BMI: Body mass index. BMI = weight/height ^2^

WHR: Waist-to-hip ratio. WHR = waist circumference/hip circumference, with higher scores indicating concentric obesity. RASM: Relative appendicular skeletal muscle. RASM = ASM/height ^2^. CCI: Charlson Comorbidity Index. A weighted index that takes into account the number and seriousness of comorbid diseases, with higher scores indicating a higher risk of death from comorbid disease. SARC-F: Simple Questionnaire to Rapidly Diagnose. SARC-F scores range from 0 to 10, and scores greater than 4 indicate a greater risk of sarcopenia. ADL: Activities of Daily Living Scale (Lawton). ADL (Lawton) scores range from 14 to 56, with higher scores indicating poorer basic activities of daily living functioning. MNA: Mini Nutritional Assessment. The MNA test is composed of simple measurements and brief questions, and the total score distinguishes between elderly patients with (1) adequate nutritional status, MNA > or = 24; (2) protein-caloric malnutrition, MNA < 17; and (3) at risk of malnutrition, MNA between 17 and 23.5.

We performed ROC analysis to test whether IFNγ/GCSF/TNFα could be used as markers for frailty screening. The AUC results indicated that the GCSF had greater sensitivity and lower specificity than IFNγ and TNFα in discriminating frail and prefrail patients from nonfrail patients ( Figure 2F). Compared with a single inflammatory factor, the combined strategy (IFNγ&GCSF, IFNγ&TNFα, and GCSF&TNFα) showed increased specificity but not sensitivity ( Figure 2F,G). Compared with a single factor, a combination of any two factors resulted in increased sensitivity ( Figure 2G). For the frail vs prefrail groups, a combined strategy (IFNγ&GCSF, IFNγ&TNFα, and GCSF&TNFα) increased sensitivity but not specificity ( Supplementary Figure S3D,E). However, almost all the combinations presented AUC values less than 0.8 ( Figure 2G and Supplementary Figure S2D,E). Although combining inflammatory factors greatly improved the specificity and sensitivity of frailty screening, the AUC values still did not reach the standard of clinical use (higher than 0.8).

We attempted to combine the GCSF with the Fried phenotype to further improve the sensitivity of serum screening for frailty and prefrailty. Interestingly, among the combinations of GCSF and the five Fried phenotype items, only the GCSF + fatigue combination had the highest sensitivity ( Figure 2H). In addition, the AUC of GCSF + fatigue (AUC = 0.92) was obviously greater than that of GCSF + lower grip strength (AUC = 0.59), GCSF + lower weight (AUC = 0.57), GCSF + lower activity (AUC = 0.71), and GCSF + lower gait speed (AUC = 0.87). These results suggest that the combination of the serological factor GCSF and the Fried phenotype item fatigue can improve the sensitivity and specificity of candidate methods for frailty and prefrailty screening.

Owing to the limitations of clinical scale-based diagnostic methods, scientists are attempting to develop new screening tools. Although metabolomics [ 8, 9] and blood proteomics [10] have promoted the screening of molecular markers of frailty in recent years, no highly sensitive or specific markers have been identified for frailty. However, frailty and normal aging share chronic inflammation, making the use of inflammatory factors as diagnostic markers difficult. Our results confirmed that frail and normal aged individuals have different inflammatory niches ( Figure 1). These findings indicate that inflammatory factors have the potential to act as molecular markers. Our study revealed that the use of a single serum marker, such as IFNγ/GCSF/TNFα, is far from sensitive or specific enough for clinical use ( Figure 2F). Despite the improvement in sensitivity and specificity shown by the combination of multiple molecular markers, this improvement is not ideal ( Figure 2G). However, the combination of a phenotype indicator (fatigue) with a molecular marker (GCSF) greatly improved the sensitivity and specificity of screening ( Figure 2H).

We initially planned to use molecular markers to address the problems of current scale-based screening or diagnostic methods. However, we found that frail and normal aged individuals have different inflammatory niches and that common inflammatory markers are not very effective for screening frailty. We then used combinations of molecular markers. Although these combinations showed improved specificity and sensitivity, they still fall short. Therefore, we adopted the strategy of combining molecular markers with clinical phenotypes. We obtained ideal results when fatigue was introduced to the GCSF. Our results provide new insights into the clinical diagnosis of frailty.

## Supplementary Data

Supplementary data is available at *Acta Biochimica et Biophysica Sinica* online.

## Supporting information

24796Supplementary_data
