# Supplementary material for: The combination of fatigue with the serum GCSF improves the performance of serological screening for frailty: Fatigue with GCSF improves the performance of serological screening for frailty
Source: Acta Biochim Biophys Sin (Shanghai). 2025 Feb 25;57(7):1195–8. doi: 10.3724/abbs.2025007 (PMC12367995; doi:10.3724/abbs.2025007)
Supplement: 24796Supplementary_data [file 24796Supplementary_data.docx]

**Supplementary Methods**

**Inflammatory niche analysis for normal aging and frailty**

The following databases were mainly used in the bioinformatics analysis of plasma proteomics: the human protein atlas [1], senescence-associated secretory phenotypes (SASP) data list [2], the plasma proteome data of the elderly with the largest sample size [3], and the plasma proteome data of the frail population with the largest sample size [4]. For all protein correlation analyses, the calculation results were provided in the supplementary data in which the original paper was directly used. Venn diagrams were used for secondary analysis of all data.

**Frailty screening and blood sample collection**

A total of 852 elderly people were included in the study from January 2018 to August 2018. All participants provided signed informed consent. People with communication problems, cognitive diseases, or severe physical conditions were excluded. Each participant underwent comprehensive geriatric assessments by trained and qualified staff. These assessments included demographic information collection, frailty evaluation (**Supplementary Figure S1**), and physical and body composition tests. Blood samples for the determination of inflammatory cytokines were taken from 67 participants. The study protocol was approved by the Research Ethics Committee of Huadong Hospital (2018K019) and was conducted in accordance with the Declaration of Helsinki. The flow chart of the methods is shown in **Supplementary Figure S2**.

**ELISA protocol**

All blood samples were collected on the spot by well-trained nurses. Once transported to the central laboratory of Huadong Hospital, the venous blood collection tubes were immediately centrifuged at 4°C and 2000 × *g* for 20 min. The upper serum was aliquoted and stored properly for ELISA. Inflammatory cytokines in human sera were quantified using human ELISA kits (Shanghai Xinyu Biotechnology, Shanghai, China) in accordance with the manufacturer’s protocols. The following markers were measured: IL1A, IL2, IL6, IL8, IL10, IL17, TNFα, IFNγ, GCSF, MCP2, CXCL1, CX3CL1, MMP7, and SOD1 (**Supplementary Table S1**).

**Statistical analysis**

All statistical analyses were performed with Prism v.6.0 (GraphPad Software, San Diego, USA). The results are expressed as the mean ± SEM. To assess the statistical significance of the difference between 2 groups for non-normally distributed data sets, we used the Mann-Whitney U test. For normally distributed data, student’s *t*-test was used to compare the variables in different groups. *P*<0.05 were considered statistically significant. The receiver operating characteristic curve (ROC) was used to evaluate the performance of all the screening tools.

**References**

1. Uhlen, M., L. Fagerberg, B. M. Hallstrom, C. Lindskog, P. Oksvold, A. Mardinoglu, A. Sivertsson, C. Kampf, E. Sjostedt, A. Asplund, I. Olsson, K. Edlund, E. Lundberg, S. Navani, C. A. Szigyarto, J. Odeberg, D. Djureinovic, J. O. Takanen, S. Hober, T. Alm, P. H. Edqvist, H. Berling, H. Tegel, J. Mulder, J. Rockberg, P. Nilsson, J. M. Schwenk, M. Hamsten, K. von Feilitzen, M. Forsberg, L. Persson, F. Johansson, M. Zwahlen, G. von Heijne, J. Nielsen, and F. Ponten. 2015. 'Proteomics. Tissue-based map of the human proteome', Science, 347: 1260419.
2. Oost, W., N. Talma, J. F. Meilof, and J. D. Laman. 2018. 'Targeting senescence to delay progression of multiple sclerosis', J Mol Med (Berl), 96: 1153-66.
3. Lehallier, B., D. Gate, N. Schaum, T. Nanasi, S. E. Lee, H. Yousef, P. Moran Losada, D. Berdnik, A. Keller, J. Verghese, S. Sathyan, C. Franceschi, S. Milman, N. Barzilai, and T. Wyss-Coray. 2019. 'Undulating changes in human plasma proteome profiles across the lifespan', Nat Med, 25: 1843-50.
4. Sathyan, S., E. Ayers, T. Gao, S. Milman, N. Barzilai, and J. Verghese. 2020. 'Plasma proteomic profile of frailty', Aging Cell, 19: e13193.


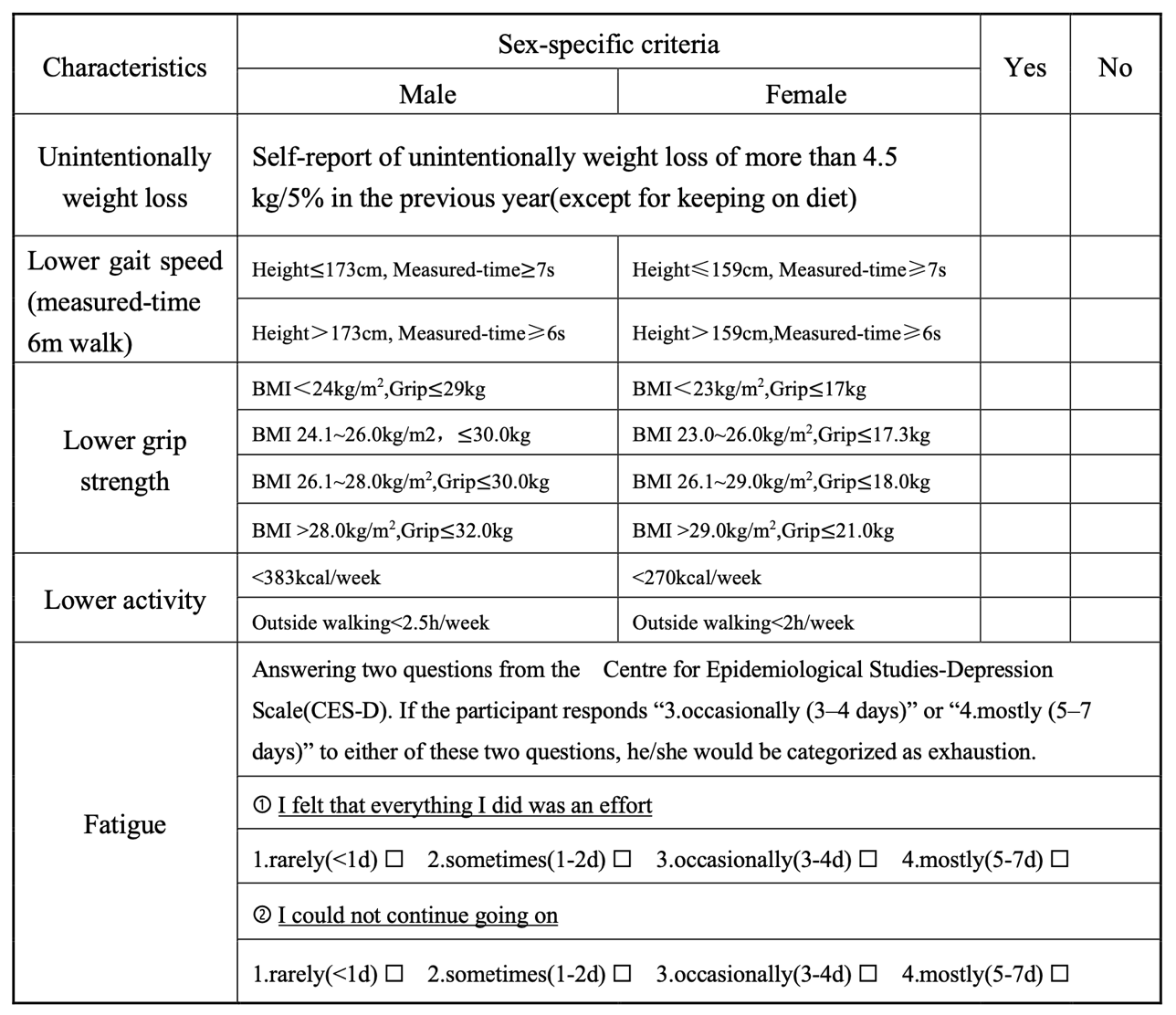


| **Supplementary Figure S1. Definition of fried phenotype (FP)** Study participants who met three or more of five characteristics were classified as frailty; meeting one or two were classified as prefrail; and those meeting none of the characteristics were classified as robust. |
| --- |
|  |


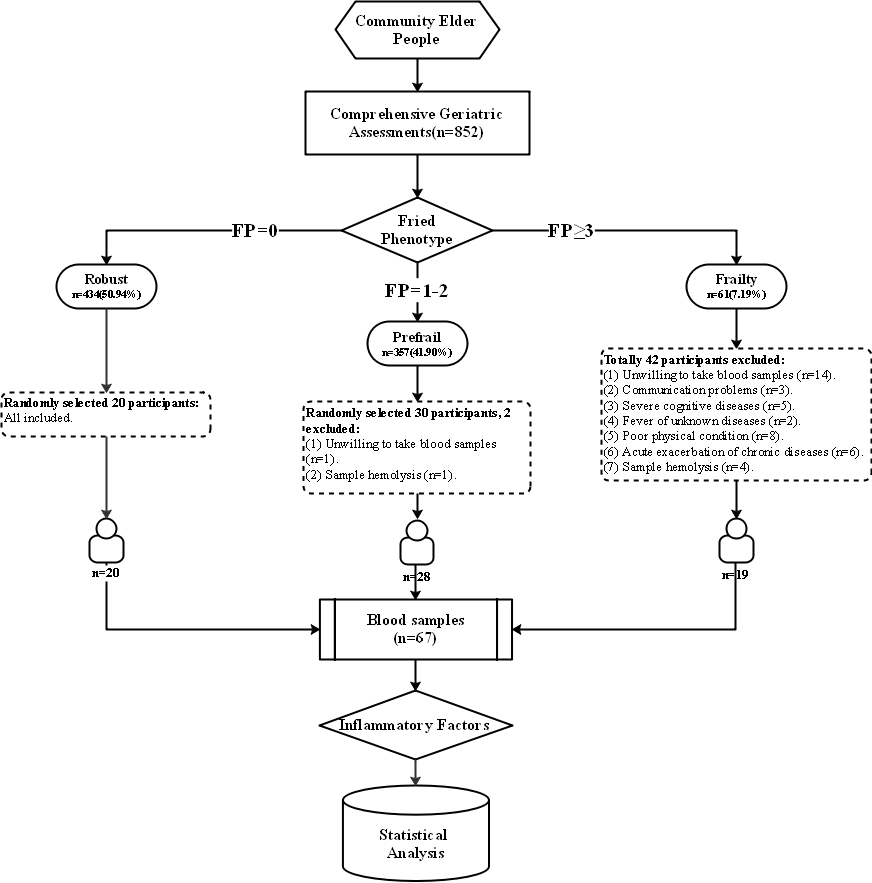


**Supplementary Figure S2. The flow chart of the clinical investigation**


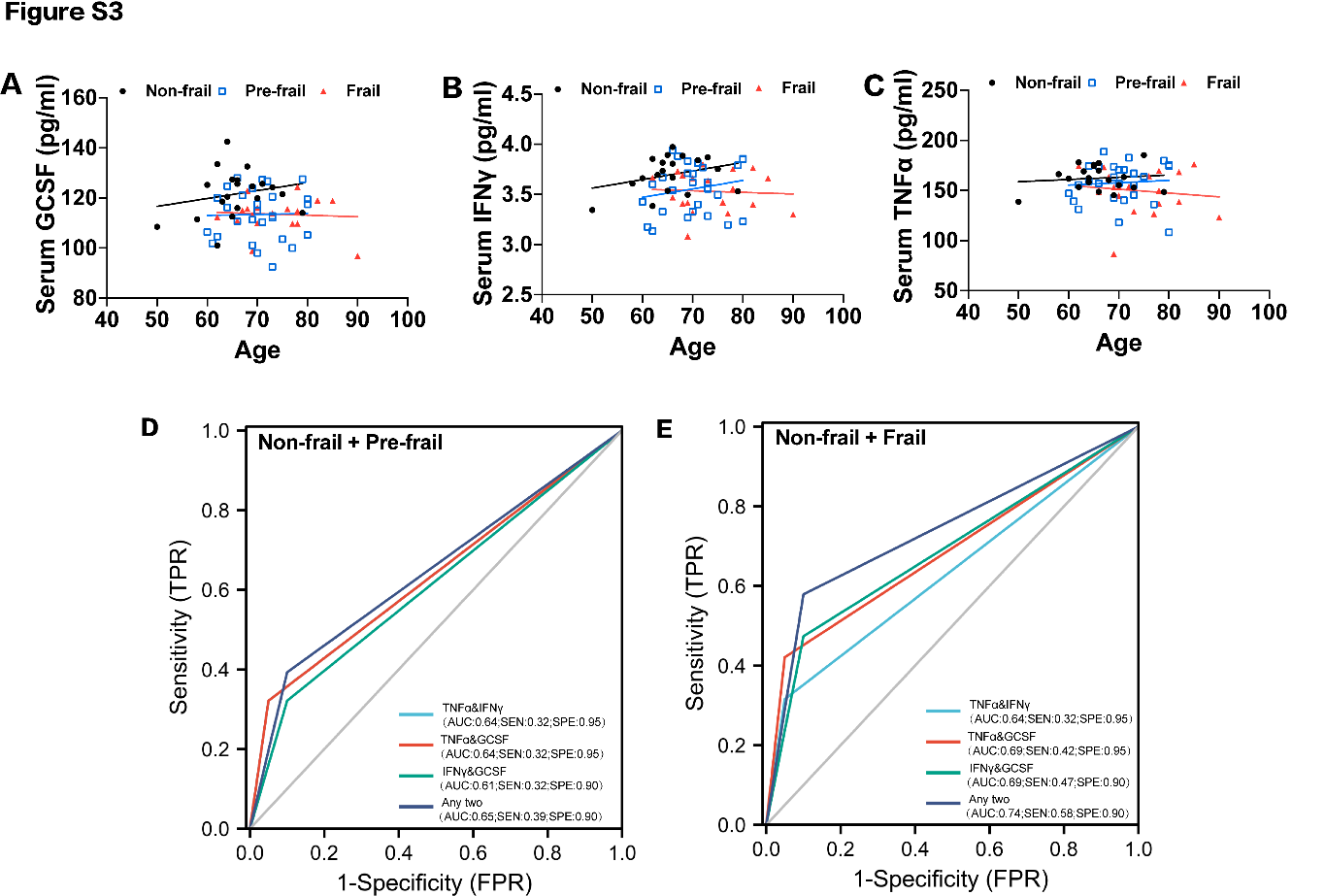


**Supplementary Figure S3. Assessment of inflammation-related factors as diagnostic markers for frailty** (A) GCSF expression decreased with increasing age in the frailty group. (B) IFNγ expression decreased with increasing age in the frailty group. (C) TNFα expression decreased with increasing age in the frailty group. (D) Any two combinations of IFNγ, GCSF, and TNFα had limited sensitivity for prefrailty screening. (E) Any combination of IFNγ&GCSF, IFNγ&TNFα, and GCSF&TNFα could significantly improve the sensitivity for frailty screening.

| **Supplementary Table S1. Efficiencies of ELISA kits** | | |
| --- | --- | --- |
| **Inflammatory cytokine** | **Sensitivity** | **Detection range** |
| Human Interleukin-1α (IL1α) | 1.9 pg/mL | 0−1000 pg/ mL |
| Human Interleukin-2 (IL2) | 1.9 pg/mL | 0−1000 pg/ mL |
| Human Interleukin-6(IL6) | 0.31 pg/mL | 0−200 pg/ mL |
| Human Interleukin-8 (IL8) | 0.78 pg/ mL | 0−500 pg/ mL |
| Human Interleukin-10 (IL10) | 0.039 pg/ mL | 0−50 pg/ mL |
| Human Interleukin-17 (IL17) | 0.78pg/ml | 0−400 pg/ mL |
| Human Tumor necrosis factor α (TNFα) | 1.9 pg/ mL | 0−1000 pg/ mL |
| Human Interferon γ (IFNγ) | 0.015 pg/ mL | 0−125 pg/ mL |
| Human Granulocyte colony stimulating factor (GCSF) | 3.9 pg/mL | 0−2000 pg/ mL |
| Human Monocyte chemotactic protein2 (MCP2) | 2.5 pg/mL | 0−1000 pg/ml |
| Human Chemokine ligand 1 (CX3CL1) | 15.6 pg/mL | 0−10000 pg/mL |
| Human Chemokine ligand 10 (CXCL10) | 3.1 pg/mL | 0−2000 pg/ mL |
| Human Matrix metalloproteinase 7 (MMP7) | 7.8 pg/mL | 0−4000 pg/ mL |
| Human Superoxide dismutase 1 (SOD1) | 15.6 pg/ mL | 0−10000 pg/ mL |
